# Prevention of Dry Socket with Ozone Oil-Based Gel after Inferior Third Molar Extraction: A Double-Blind Split-Mouth Randomized Placebo-Controlled Clinical Trial

**DOI:** 10.3390/gels9040289

**Published:** 2023-04-01

**Authors:** Alberto Materni, Claudio Pasquale, Eugenio Longo, Massimo Frosecchi, Stefano Benedicenti, Matteo Bozzo, Andrea Amaroli

**Affiliations:** 1 Department of Surgical and Diagnostic Sciences (DISC), University of Genoa, 16132 Genoa, Italy; 2 Department of Civil, Chemical and Environmental Engineering (DICCA), University of Genoa, 16100 Genoa, Italy; 3 Department of Earth, Environmental and Life Sciences (DISTAV), University of Genoa, 16132 Genoa, Italy

**Keywords:** alveolar osteitis, alveolar periostitis, alveolar periostitides, alveolus, dental, wisdom tooth, mucositis, third molar, oral disease

## Abstract

Tooth extraction is followed by a sequence of elaborate local changes affecting hard and soft tissues. Dry socket (DS) can occur as intense pain around and in the extraction site, with an incidence from 1–4% after generic tooth extraction to 45% for mandibular third molars. Ozone therapy has gained attention in the medical field because of its success in the treatment of various diseases, its biocompatible properties and its fewer side effects or discomfort than drugs. To investigate the preventive effect of the sunflower oil-based ozone gel Ozosan^®^ (Sanipan srl, Clivio (VA), Italy) on DS, a double-blind split-mouth randomized placebo-controlled clinical trial was conducted according to the CONSORT guidelines. Ozosan^®^ or the placebo gel were put in the socket, and the gels were washed off 2 min later. In total, 200 patients were included in our study. The patient population comprised 87 Caucasian males and 113 Caucasian females. The mean age of the included patients was 33.1 ± 12.4 years. Ozosan reduced the incidence of DS after inferior third molar extraction from 21.5% of the control to 2% (*p* < 0.001). Concerning the dry socket epidemiology, the incidence was not significantly correlated with gender, smoking or mesioangular, vertical or distoangular Winter’s classification. Post hoc power calculation showed a power of 99.8% for this data, with alpha = 0.001.

## 1. Introduction

Tooth extraction is one of the main procedures for oral health management. However, despite being a routine surgical practice, its consequences have not always been accurately assessed. Tooth extraction induces a sequence of elaborate local changes affecting hard and soft tissues, and the healing of the extraction socket can, therefore, show complications that reduce the patient’s quality of life. One possible complication is a dry socket (DS), a postoperative problem also referred to as alveolar osteitis, fibrinolytic alveolitis, septic socket, localized osteomyelitis or necrotic socket [1]. A DS manifests as intense pain around and in the extraction site, 1 to 3 days after tooth extraction. Its incidence depends on the tooth extracted, with an overall incidence of 1–4% after extraction, but a 10-fold higher incidence for lower teeth than for upper teeth, and an incidence of 45% for mandibular third molars [1,2].

A considerable number of aetiological factors such as dislodgement of the clot, oral micro-organisms, trauma during surgery, anaesthesia, drugs (oral contraceptives), smoking, age and gender appear to influence DS although a multifactorial origin is likely [3,4].

Because of the inconsistent identification of the main causative variables of DS, there is no consensus on the best prophylactic management for this disorder. The literature suggests the identification of risk factors associated with prevention, and both non-pharmacological and pharmacological measures can support DS aetiology and recovery [2,3,5,6].

However, despite the advice for DS prevention, including procedural details and surgical skills, no single method has achieved universal success or acceptance [2].

Past and recent reviews [2,3] have described the therapeutic interventions currently used in DS management, which can include antibacterial, antiseptic, antifibrinolytic and clot support agents, lavages and obtundent dressings. Management is generally directed primarily towards timely relief of the patient’s pain during the healing process through palliative means (antibacterial or obtundent dressings, topical anaesthetic or combinations of these) [2,7,8].

Ozonated compounds have been employed in industries and have recently gained attention in the literature for clinical applications [9,10]. Indeed, ozone therapy has gained attention in the medical field because of its success in the treatment of various diseases, its biocompatible properties and its fewer side effects or discomfort than drugs [11]. In particular, its immunomodulation, analgesic, antihypnotic, detoxifying, antimicrobial, bioenergetic and biosynthetic properties could support many dentistry challenges [12].

It is worth noting that Scribante et al. [13] have recently highlighted the significance of tailoring minimally invasive protocols to manage the oral microbiota, clinically and microbiologically, with ozone playing a crucial role among other approaches. This can help integrate bacterial load, increase the proportion of commensal bacteria, prevent the resetting of the oral microbiota and avoid the use of chemical pharmacological substances. 

The subject of ozone and its uses in medicine is still a surprising and controversial topic of discussion [14].

As reviewed by Saini [12], ozone therapy can be applied in three fundamental forms: ozonated water, ozonated olive oil in natural and gel formulations and oxygen/ozone gas. Although the properties of ozone may be similar in the different formulations, the gas’s stability, release and effect vary with variations in the product’s gaseous, aqueous, oily or gel condition [9]. Indeed, gaseous ozone can be toxic and unstable, and ozonated water decomposes more rapidly than gels [9,15,16]. Therefore, ozone in the form of an oil-based gel may be significantly more stable when in contact with the target surface for a more extended period [17]. 

Recently, a novel sunflower oil-based ozone gel (Ozosan^®^—Sanipan srl, Clivio (VA), Italy) showed the efficacy of ozone in the healing of osteoradionecrosis of the jaws [15] and as a dentin primer, to improve the microtensile bond strength and endogenous enzymatic activity of two adhesive systems [18]. 

In the current study, we investigated the preventive effect of this oil-based ozone gel on DS in a double-blind split-mouth randomized placebo-controlled clinical trial conducted according to the CONSORT guidelines.

The predictor variable was the property of ozone in the sunflower oil-based gel formula [12,14,17]. The primary endpoint was the reduction in DS incidence after an inferior third molar extraction. The secondary endpoint was the adverse effects of ozone.

## 2. Results

### 2.1. Patient and Baseline Characteristics

**Figure 1:**
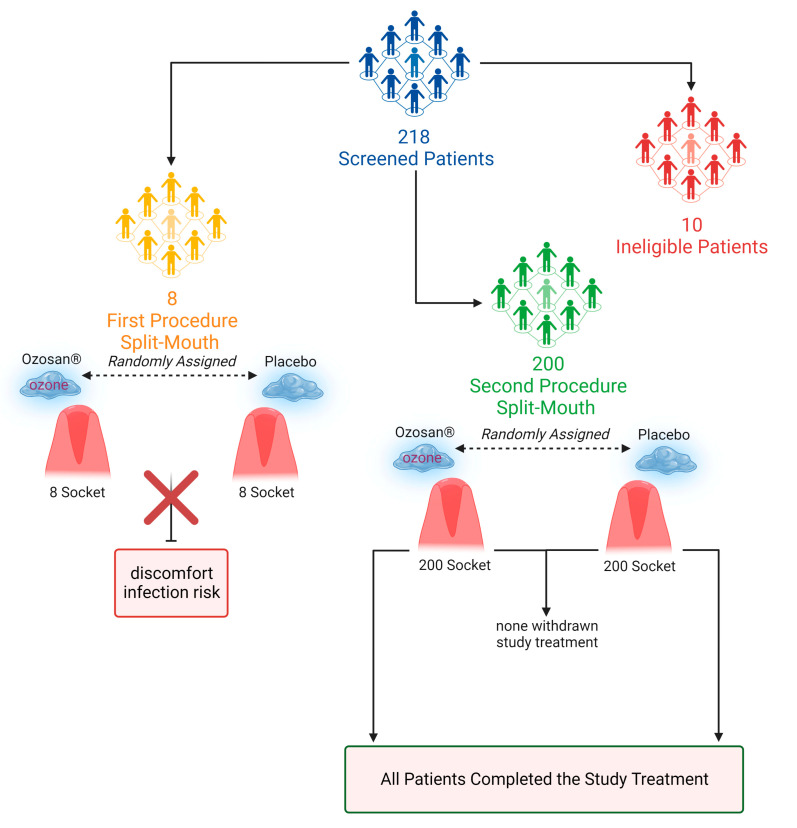
Design of experimental set-up according to CONSORT guidelines.

More than 200 patients (Figure 1) with two impacted lower third molars were assessed and treated. Eight patients were excluded due to the failure of the first procedure, which was followed by discomfort and infection risks as a consequence of the trapping of food by the gels.

The 200 remaining patients received the second procedure and were followed to monitor the occurrence of DS and the side effects of ozone. 

As shown in Table 1, the patient population comprised 87 Caucasian males and 113 Caucasian females. The mean age of the included patients was 33.1 ± 12.4 years (range: from 18 to 65 years). Partial inclusion of the third molar was observed in 72% of patients, while 28% showed total inclusion. Osteotomy and odontotomy were performed in all patients (100%). According to Winter’s classification of impacted third molars, 42% of patients were classified as Mesio (M), 45% as Vertical (V) and 13% as Distally (D) tilted. Of the 200 patients, 36.6% smoked cigarettes.

Patient recruitment and follow-up were completed between June 2021 and October 2022.

### 2.2. Effect of Ozonated Gel on Dry Socket Incidence

A dry socket occurred in four patients in the Ozosan group and in 43 patients in the placebo group (Table 2). The incidence percentage of dry socket was 2% and 21.5%, respectively. Chi-square analysis revealed a highly significant difference (*p* < 0.001) between the Ozosan and placebo treatments regarding the occurrence of DS (Table 2).

The post hoc power calculation showed a power of 99.8% for our data, with alpha= 0.001. No bilateral alveolar osteitis was described and complete healing of the wound was observed on both sides of the mouth (Test and Placebo side), involving complete soft tissue wound closure with correct repair/neoformation of the attached gingiva and oral mucosa. Dry socket occurred in two males and two females in the Ozosan group and in 17 males and 26 females in the placebo group. No significant differences (*p* > 0.05) were observed in relation to gender in the Ozosan vs placebo treatment (Table 3). Inferior third inferior molar inclusion Winter’s Classification (Table 4) and smoking (Table 5) were not correlated with the DS emergence (*p* > 0.05). 

### 2.3. Amount of Peroxide in Ozosan^®^ Gel and Release

**Figure 2:**
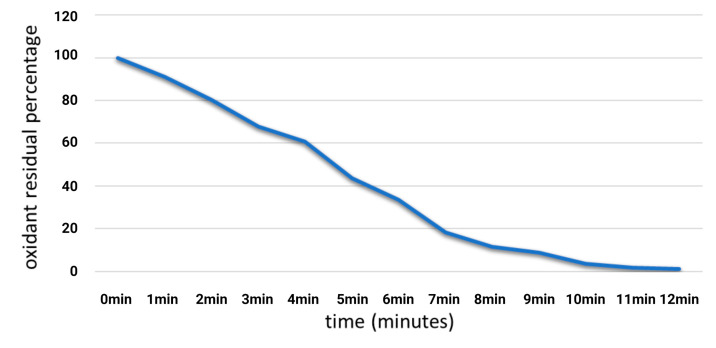
Percentage of Ozosan’s ozone release over time. According to the UNI EN ISO 3960:2017 standards, the total amount of peroxide in the gel was evaluated at 1322.4 meqO2/Kg (100%). The time-temperature-dependent release expressed in % indicates that after 10 min, about 97% of the initial peroxide had been released. Of note, at the same 2 min exposure time employed in our study, less than 20% of the peroxide had been released.

According to the UNI EN ISO 3960:2017 standards, the percentage of Ozosan’s ozone release over time was evaluated. The total amount of peroxide in the gel was found to be 1320 meqO2/Kg (100%), which meets with the manufacturer’s instructions. The time- and temperature-dependent release expressed in percentage is shown in Figure 2. Essentially, the % of oxidizing residues in the gel was, 100% (0 min), 91.10% (1 min), 80.10% (2 min), 67.70% (3 min); 60.70% (4 min), 43,70% (5 min), 33.50% (6 min), 18.20% (7 min), 11.40% (8 min), 8.70% (9 min), 3.50% (10 min), 1.6% (11 min), <1.5% 12 min. It is worth noting that, in our study, at the same 2 min exposure time, less than 20% of the peroxide had been released. These results highlight the significant difference in peroxide release between the 2 min exposure time and the 10 min exposure time. The lesser exposure time resulted in a lower peroxide release percentage, which could have important implications for the role and cellular responses to Ozosan in certain applications.

## 3. Discussion

Epidemiological studies have revealed several contradictory risk factors in the development of post-extraction alveolitis: surgical trauma as a consequence of the difficulty in extraction and/or inexperience of the surgeon, inadequate intraoperative irrigation, use of oral contraceptives and/or immunosuppression, advanced age, female sex and smoking. However, the true causes of the condition are not known [2,7,19,20,21].

Our data showed a dry socket prevalence of 21.5%, consistent with its more frequent occurrence following inferior third molar extraction [2,19]. This incidence was lower than the 35–45% reported in the literature [1,7,8] but consistent with the data of Kaur et al. [20] from 150 patients. 

While DS epidemiology was not prominent in our study, its incidence was not significantly correlated with gender, smoking or mesioangular, vertical or distoangular Winter’s classification. As discussed by Pattanaik et al. [11] and Parthasarathi et al. [22], the smoking data were supported by recent research showing that smoking has no effect on the incidence of alveolar osteitis, particularly when the results were analyzed in a multivariate fashion [21]. The lack of any gender correlation is also consistent with Parthasarathi et al. [22], who found no difference in the incidence of alveolar osteitis between males and females, and no influence of the Winter classification, which can be attributed to the long-standing experience of the surgeons [23].

Conversely, the ozone in the sunflower oil-based gel formula (Ozosan) significantly reduced the occurrence of DS on the treated side of the mouth. Our double-blind split-mouth randomized placebo-controlled clinical trial showed a DS incidence after a third inferior molar extraction of 2% on the Ozosan-treated side and a more than tenfold higher incidence on the placebo side. Comparison with the results obtained by Haraji et al. [24] and Kaur et al. [20] when using chlorhexidine and a similar experimental setup (80 and 150 patients, respectively) revealed the significantly more efficient effect of Ozosan (*p* < 0.05); no significant differences were detected among the corresponding placebo groups. The use of chlorhexidine can result in discomforts such as mucosal irritation, altered taste sensation, tooth staining and possible impairment of wound healing, which have discouraged prolonged treatment for the irrigation of oral mucosa or the periodontal pockets [25,26]. Conversely, ozonated compounds show higher biocompatibility [27]. The evidence-based literature shows the undisputed disinfection power of ozone, which, at a concentration of 4 mg/L in water, effectively killed gram-positive and gram-negative oral bacteria and fungi [28]. However, according to the time-dependent release of ozone from the Ozosan gel shown in our results, a correlation between antimicrobial ozone properties and DS was only apparent following the first (aborted) procedure, which involved longer application times and, therefore, a higher gas release. Indeed, 10 min is required to release almost the entire amount of gas and induce a bactericidal effect. Conversely, after 2 min, less than 20% had been released. The few minutes of Ozosan application in the second (reliable) procedure was probably not sufficient to disinfect the surgery-impacted area. However, the results suggest a stimulating low-ozone-dose effect on tissues. Indeed, gases such as reactive oxygen species (ROS) and nitric oxide (NO) drastically improved healing recovery [29,30], and novel biomaterials that are able to release these gases have been developed [31,32].

Recently, Viebahn-Haensler and León Fernández [33] reviewed the low-dose ozone theory and its mechanisms of action on diseases. Essentially, they described the bioregulatory role of low-dose ozone in chronic inflammatory diseases and its direct mechanism of action in topical treatments via radical reactions.

Previous studies on stomatological challenges [34] have described the efficacy of ozonated compounds on osteoradionecrosis of the jaws [17], anti-inflammatory effects [35], and post-surgical pain [36,37], as well as ozonated water, which appeared to be effective adjuvant treatment to scaling and root planning for periodontal disease [13]. Based on the excellent clinical responses of several diseases and pain to ozone, the biological activity of this gas would be properly mediated by modulated responses to mild oxidative stress induced after its application [14]. Thus, as suggested by Re [14], according to the ancestral environment of our cells and its strictly metabolic binding to oxygen burn, the ubiquitous signalling pathway mediated by NF-E2–related factor 2 (Nrf2) could play a role in the low-dose ozone-mediated effects. Indeed, the Nrf2 pathway acts in the defence against oxidative stress, carcinogenesis, anti-inflammation, stem cell regulation, anti-ageing, protection against brain and skin injuries and so forth [38]. Overall, our data seem to suggest a lower prevalence of DS following Ozosan treatment, which may indicate the preventive effect of ozone on the signalling pathways that lead to osteoblast apoptosis, inflammation and tissue necrosis, factors that, as reported by Mamoun [8], appear strictly correlated with DS incidence.

## 4. Conclusions

In conclusion, our double-blind split-mouth randomized placebo-controlled clinical trial supported our primary and secondary endpoint. Indeed, Ozosan reduces the incidence of DS after an inferior third molar extraction. Additionally, no side effects or macroscopic differences in post-extraction wound recovery were observed. Focused in vitro and in vivo studies on the effect of Ozosan on the regeneration of tissues will be necessary to clarify the possibility of combining DS prevention with more rapid recovery from surgery. This promising preventive result on alveolitis paves the way for further randomized studies on the treatment of periodontitis and inflammatory oral diseases.

## 5. Materials and Methods

### 5.1. Study Design and Participants

Our double-blind split-mouth randomized placebo-controlled clinical trial followed the CONSORT guidelines (Figure 1 and Figure S1) and was approved by the University of Genoa, Department of Surgical Sciences and Integrated Diagnostics (DISC) Ethics Committee (decision number: document No. 2021/55 of the University Research Ethics Committee). The study was conducted in accordance with the Declaration of Helsinki, Good Clinical Practice guidelines of 1975, as revised in 2013, and applicable local laws. The procedures were explained to participants who signed a consent form.

Eligible patients were females or males who required bilateral extraction of mandibular third molars. Patients were excluded if they presented acute swelling, infections, diabetes, cancer, genetic immunodeficiency, angiogenic disorders or any neurological or psychological diseases, and if they had taken anti-inflammatory, antibiotic, bisphosphonate, analgesics and/or blood pressure or angiogenic medications 3 months before enrolment. Pregnant and lactating women were also excluded. A full medical history of each subject was obtained, and a thorough oral examination was undertaken before participation in the study.

### 5.2. Randomization and Masking

Each patient’s right and left inferior third molars were randomly allocated (1:1) to be treated with either novel sunflower oil-based ozone gel (Ozosan^®^, Sanipan srl, Clivio (VA), Italy) (O group) or placebo gel (P group). Randomization was based on a random–sequence software program (www.random.org/sequences (accessed on 20 February 2023)), which generates randomized sequences. The molar was added to the O group if it received an odd number, and to the P group if it received an even number.

### 5.3. Operative Technique

A traditional envelope flap approach was performed [39,40,41]. This classical technique consists of a first distal incision starting from the 2M distal surface in the attached gingiva and moving distobuccally 45° for around 10 mm. Then a second incision is made perpendicularly to the first incision around 3 or 4 mm distally from the 2M, directed towards the 2M buccal sulcus, going through it and finishing in the buccal papilla between the second and first molars. A triangle of soft tissue is defined just distal to the 2M, which is eliminated with a mini-Friedmann 90° rongeur (RMF90 rongeur Friedmann 90°, small cod RMF90, Hu-Friedy Mfg. Co., Chicago, IL, USA). A full-thickness flap is elevated using a Prichard periosteal elevator (3 Prichard periosteal PPR36, Hu-Friedy Mfg. Co., Chicago, IL, USA) exposing the buccal and the occlusal bone in the case of fully impacted teeth to the oblique mandibular line. The flap is protected on the buccal side with an 11 × 40 mm Langenbeck metal retractor (Stoma Dentalsysteme Dentalsysteme GmbH & Co KG, Emmingen-Liptingen, Germany). A buccal ostectomy is performed using a surgical steel bur (Komet H31LR316 016, Komet Dental Gebr. Brasseler GmbH & Co., Lemgo, Germany) in a 45° angle surgical hand-piece with a cooling water port and no air spray (NSK TI-MAX 45° Stand-Titan, NSK Dental, Kanuma, Japan). An odontotomy is made in order to extract the tooth more efficiently using a surgical hart-metal bur (Komet H31LR316 016, Komet Dental Gebr. Brasseler GmbH & Co., Lemgo, Germany) in a 45° angle surgical handpiece with a cooling water port and no air spray (NSK TI-MAX 45° Stand-Titan, NSK Dental, Kanuma, Japan). The tooth is extracted in sections to avoid a wider ostectomy. Friedman elevators 31F, 32F and 3C are used to assist extraction (Hu-Friedy Mfg. Co., Chicago, IL, USA). The extraction site is evaluated using a 3 mm Lucas bone curette, which is kept superficial to avoid any accidental damage to the inferior alveolar nerves. Care is taken to avoid placing the instrument too deeply into the apical portion of the alveolus. The surgical procedure concludes with the placement of a monofilament synthetic absorbable poliglecaprone 4/0 USP surgical PGCL suture, using a 16 mm 3/8 OMNIA reverse cutting needle (OMNIA S.p.A., Fidenza, Italy). This helps to maintain the flap in its original position, without tension, and to create a triangular open communication distal to the 2M for an open healing process. A knot is tied to hold the flap distal to the 2M in an apical position, and usually, two additional knots are tied to close the distal incision.

### 5.4. Ozone Gel or Placebo Treatment

To test the effect of ozone gel on the occurrence of DS, the Ozosan^®^ Gel was employed. According to the manufacturer’s description, the gel has a sunflower oil base that has undergone ozone saturation treatment and been gelled; the cold gel (5 °C) formulation prevents rapid ozone migration towards the surface. As the temperature of the gel increases due to body temperature, the capacity of the gel to hold the ozone molecules vanishes, thus generating their rapid dispersion in the air [17] (https://bioactiva.it/prodotti/ (accessed on 20 February 2023)). The placebo gel was a similar-looking sunflower oil without ozone. Therefore, the patients and study team members responsible for the administration of the treatment were masked in treatment allocation. The Ozosan and placebo gels were inserted in the socket before suturing. One millilitre of gel was necessary to fill the socket.

In the first round of procedures, the gels were left in the socket and suturing was performed. However, patients experienced discomfort (see results section) and the procedure was subsequently modified, such that the gels were added to the socket as described above, but then washed away after 2 min, before suturing (there was no gel in the socket after suturing). Two experienced clinicians, who were not involved in the surgery or treatment administration, assessed the outcomes and showed 100% intra-examiner agreement for all DS diagnosis criteria, following the advice given by Rakhshan et al. [4]. Calibration was performed before the study following an independent assessment of the experimental purposes. Patients were consulted daily and monitored for the first week post-extraction and at 8 weeks in order to evaluate the resolution of the soft tissue wound. The two examiners were blinded to the two experimental groups. The investigator responsible for statistical data analysis was also masked to treatment assignment. An anonymously numbered questionnaire was used to gather patients’ screening information.

### 5.5. Outcomes

In accordance with the literature [12,14,17], the predictor variable was the property of ozone in the oil-based gel formula. The primary endpoint was the reduction of DS incidence after an inferior third molar extraction. The secondary endpoint was the adverse effects of ozone.

### 5.6. Determination of Peroxide Values in Ozosan^®^ Gel

The peroxide amount was evaluated through iodometric determination in accordance with the UNI EN ISO 3960:2017 standards [42] and according to the classical procedure described by Radzimierska-Kaźmierczak et al. [43], modified. The release was evaluated to mimic the experimental conditions for 12 min. The release of peroxide was determined and expressed in milliequivalents of active oxygen per kilogram of gel, (methods error: ±0.2 mEq O2/kg).

### 5.7. Data Analysis

Outcome measures were statistically analyzed. The incidence (yes/no) of DS and the sample size’s statistical power were considered. Calculations were performed using SPPS 25 (IBM Corp. Released in 2017. IBM SPSS Statistics for Windows, Version 25.0, Armonk, New York, NY, USA). Tests were considered significant if *p* < 0.05.

According to Rosner, the sample size was calculated to determine the minimum number of subjects for adequate study power using the ClinCalc Sample Size Calculator [44]. The previously published mean value of the primary endpoint and the work of Kaur et al. were taken into account. Therefore, based on an incidence of 22.6% in group 1 and 6.6% in group 2, a sample size of 150 patients was considered sufficient to have a power of 0.8 with an alpha of 0.5 and a beta of 0.2.

## Figures and Tables

**Table 1:** Baseline characteristics of the treated population.

Patients’ Age	(Mean ± Standard Deviation (Min-Max))			
	33.1 ± 12.4 (18–64)			
Patients’ gender	Male	Female		
	87	113		
Smoker	Yes	No		
	36.60%	63.40%		
Winter classification of the third molar	Vertical	Mesioangular	Distoangular	Horizontal
	45%	42%	13%	0%

**Table 2:** The table displays the number of diagnosed dry sockets after treatment with ozone in the form of sunflower oil-based gel (Ozosan) or placebo gel. The incidence percentage of dry sockets was calculated with respect to 200 inferior third molars extracted per treatment. The * symbol denotes a statistically significant difference (*p* < 0.05) between the two treatments, as calculated using the chi-square test.

Dry Sockets Diagnosis	Ozosan	Placebo
Presence of dry socket (number)	4	43
Absence of dry socket (number)	196	157
% of diagnosed dry sockets	2% *	21.5% *

**Table 3:** The table displays the number of diagnosed dry sockets in female or male patients after treatment with ozone in the form of sunflower oil-based gel (Ozosan) or placebo gel. The incidence percentage of dry sockets was calculated with respect to the number of female patients (113) or male patients (87) with third molars extracted. The * symbol denotes a statistically significant difference (*p* < 0.05) between the two treatments, as calculated using the chi-square test.

Treatment with Ozone in the Form of Sunflower Oil-Based Gel (Ozosan)		
	Males	Females
Presence of dry socket (number)	2	2
Absence of dry socket (number)	85	111
% of diagnosed dry sockets	2.30%	1.80%
Treatment with placebo gel		
	Males	Females
Presence of dry socket (number)	17	26
Absence of dry socket (number)	70	87
% of diagnosed dry sockets	19.50%	23.00%

**Table 4:** The table displays the number of diagnosed dry sockets with respect to the third molar Winter’s Classification after treatment with ozone in the form of sunflower-oil-based gel (Ozosan) or placebo gel. The incidence percentage of dry sockets was calculated with respect to the number of mesioangular (84), vertical (88) or distoangular (28) classified third molars. The * symbol denotes a statistically significant difference (*p* < 0.05) between the two treatments, as calculated using the chi-square test.

Treatment with Ozone in the Form of Sunflower Oil-Based Gel (Ozosan)			
	Mesioangular	Vertical	Distoangular
Presence of dry socket (number)	2	1	1
Absence of dry socket (number)	82	87	27
% of diagnosed dry sockets	2.40%	1.10%	3.60%
Treatment with placebo gel			
	Mesioangular	Vertical	Distoangular
Presence of dry socket (number)	21	14	8
Absence of dry socket (number)	63	74	20
% of diagnosed dry sockets	25.00%	15.90%	28.60%

**Table 5:** The table displays the number of diagnosed dry sockets in smoker and non-smoker patients after treatment with ozone in the form of sunflower-oil-based gel (Ozosan) or placebo gel. The incidence percentage of dry sockets was calculated with respect to the number of female patients (113) or male patients (87) with third molars extracted. The * symbol denotes a statistically significant difference between the two treatments, as calculated using the chi-square test.

Treatment with Ozone in the Form of Sunflower-Oil-Based Gel (Ozosan)		
	Smokers	Non-Smokers
Presence of dry socket (number)	2	2
Absence of dry socket (number)	71	125
% of diagnosed dry sockets	2.70%	1.60%
Treatment with placebo gel		
	Males	Females
Presence of dry socket (number)	20	23
Absence of dry socket (number)	53	104
% of diagnosed dry sockets	27.40%	18.10%

## Data Availability

All data generated or analyzed during this study are included in this published article (and its Supplementary Information files).

## Supplementary Materials

The following supporting information can be downloaded at: https://www.mdpi.com/article/10.3390/gels9040289/s1, Figure S1: CONSORT checklist.
