# Supplementary material for: Prevention of Dry Socket with Ozone Oil-Based Gel after Inferior Third Molar Extraction: A Double-Blind Split-Mouth Randomized Placebo-Controlled Clinical Trial
Source: Gels. 2023 Apr 1;9(4):289. doi: 10.3390/gels9040289 (PMC10137763; doi:10.3390/gels9040289)
Supplement: Supplementary file 1 [file gels-09-00289-s001.zip › gels-2268901-supplementary.pdf]

CONSORT 2010 checklist of information to include when reporting a randomised trial*

Section/Topic	Item No	Checklist item	Reported on page No
**Title and abstract**			
	1a	Identification as a randomised trial in the title	PAG 1
	1b	Structured summary of trial design, methods, results, and conclusions (for specific guidance see CONSORT for abstracts)	PAG 1
**Introduction**			
Background and objectives	2a	Scientific background and explanation of rationale	PAG 1-2
	2b	Specific objectives or hypotheses	PAG 2-3
**Methods**			
Trial design	3a	Description of trial design (such as parallel, factorial) including allocation ratio	PAG 3
	3b	Important changes to methods after trial commencement (such as eligibility criteria), with reasons	PAG 5-6
Participants	4a	Eligibility criteria for participants	PAG 3
	4b	Settings and locations where the data were collected	PAG 3
Interventions	5	The interventions for each group with sufficient details to allow replication, including how and when they were actually administered	PAG 4-6
Outcomes	6a	Completely defined pre-specified primary and secondary outcome measures, including how and when they were assessed	PAG 6
	6b	Any changes to trial outcomes after the trial commenced, with reasons	NOT APPLICABLE
Sample size	7a	How sample size was determined	PAG 6
	7b	When applicable, explanation of any interim analyses and stopping guidelines	NOT APPLICABLE
Randomisation:			
Sequence generation	8a	Method used to generate the random allocation sequence	PAG 4
	8b	Type of randomisation; details of any restriction (such as blocking and block size)	PAG 4
Allocation concealment mechanism	9	Mechanism used to implement the random allocation sequence (such as sequentially numbered containers), describing any steps taken to conceal the sequence until interventions were assigned	PAG 4
Implementation	10	Who generated the random allocation sequence, who enrolled participants, and who assigned participants to interventions	PAG 4
Blinding	11a	If done, who was blinded after assignment to interventions (for example, participants, care providers, those assessing outcomes) and how	PAG 6
	11b	If relevant, description of the similarity of interventions	NOT

CONSORT 2010 checklist *Page 1*

Figure S1. CONSORT cheklist.
